# Extraosseous Ewing’s Sarcoma of the Parapharyngeal Space - A Rare Entity - with Review of Literature

**Published:** 2019-01

**Authors:** Divya Khosla, Shalini Verma, Rajpal S Punia, Arjun Dass, Kislay Dimri, Gurbir Kaur, Awadhesh K Pandey

**Affiliations:** 1 *Department of Radiotherapy, Government Medical College and Hospital, Chandigarh, India.*; 2 *Department of Pathology, * *Government Medical College and Hospital, Chandigarh, India.*; 3 *Department of Otorhinolaryngology and Head and Neck Surgery, Government Medical College and Hospital, Chandigarh, India.*

**Keywords:** Ewing’s sarcoma, Extraosseous, Head and neck, Parapharyngeal spaceP prognosis

## Abstract

**Introduction:**

Extraosseous Ewing’s sarcoma (EES) of the head and neck region is a rare occurrence, and Ewing’s sarcoma of the parapharyngeal space is even rarer. To the best of our knowledge, only three cases of EES of the parapharyngeal space have been reported in the literature.

**Case Report:**

We report a rare case of EES of the parapharyngeal space in an 8-year-old girl. She presented with complaints of earache, difficulty in breathing and swallowing and bleeding from the mouth. Investigations revealed a large parapharyngeal mass causing narrowing of the nasopharyngeal and oropharyngeal airway with skeletal and lung metastasis. Biopsy from the parapharyngeal mass was suggestive of malignant small round cell tumor. The patient was treated with chemotherapy and radiotherapy, but developed brain metastasis and succumbed to disease approximately 1 year after diagnosis. Herein, we describe the characteristic clinicopathological features and treatment with a comprehensive review of the literature.

**Conclusion:**

EES in this unusual location behaves aggressively, with a high rate of recurrence and distant metastasis. Aggressive multimodal treatment comprising of multi-agent chemotherapy, surgical resection if feasible, and radiotherapy should be considered.

## Introduction

Extraosseous Ewing sarcoma (EES) is histologically and molecularly similar to Ewing sarcoma of the bone. These neoplasms are composed of small round blue cells of neuroectodermal origin, and about 85% of Ewing sarcomas are associated with translocation t(11;22)(q24.1;q12.2) (1). EES is an uncommon neoplasm with aggressive behavior that can develop in the soft tissues at any location. It may rarely arise in the head and neck region and accounts for 4–9% of all Ewing sarcomas (2–5). Literature on head and neck sarcomas is scarce, consisting of a few retrospective series and case reports. The most common sites of presentation are the skull, mandible and maxilla, followed by the orbit, nasal cavities and paranasal sinuses (3,6,7). EES usually follows an aggressive course with a high incidence of local recurrence and distant metastasis. The treatment of Ewing sarcoma of the head and neck region is challenging due to the rapid involvement of closely related tissue planes and its close proximity to vital structures, which makes it difficult to achieve negative surgical margins. The EES of the parapharyngeal space is extremely rare, with only three case reports published till date. We herein report fourth case of EES in the parapharyngeal space, documenting detailed clinical presentation, pathological features and treatment.

## Case Report 

**Fig1:**
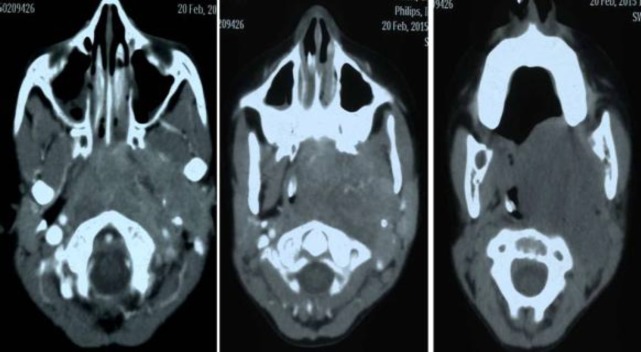
CECT showing a large heterogeneous enhancing soft tissue density mass in the left parapharyngeal space extending to the oropharynx, laryngopharynx, nasopharynx, prevertebral space, paravertebral region and superiorly reaching up to the base of the skull

An 8-year-old-girl presented with principal complaints of pain in the left ear, bleeding from the mouth, difficulty in swallowing, change in the quality of voice and difficulty in breathing since one month. There was no preceding history of trauma to the neck or associated history of nasal obstruction or epistaxis. Personal and family history was not relevant. On clinical examination, there was a large submucosal bulge present over the left tonsillar fossa which crossed the midline, pushing the uvula to the opposite side and obscuring the oropharyngeal airway. The endolarynx could not be examined. On nasal endoscopy, bilateral choanae were blocked by a pinkish friable mass obliterating the whole of the nasopharynx. There were no palpable neck nodes. Hematological, liver and renal function tests were within normal limits. Contrast enhanced computed tomography (CECT) was suggestive of a large heterogeneous enhancing soft tissue density mass in the left parapharyngeal space with extension to the oropharynx, laryngopharynx, nasopharynx, prevertebral space, paravertebral region and reaching superiorly up to the base of skull (Fig.1a,b,c). The mass was causing displacement of the left carotid artery, complete compression of the internal jugular vein and significant narrowing of the airway. 

**Fig 2:**
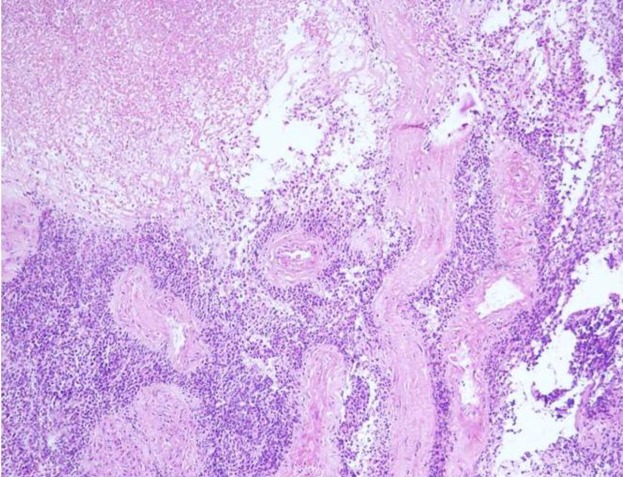
Biopsy showing malignant round tumor cells and geographic areas of necrosis (H&E × 40)

Biopsy of the mass showed sheets and nests of round-to-oval tumor cells with hyperchromatic nuclei and scant cytoplasm (Fig.2). 

**Fig 3:**
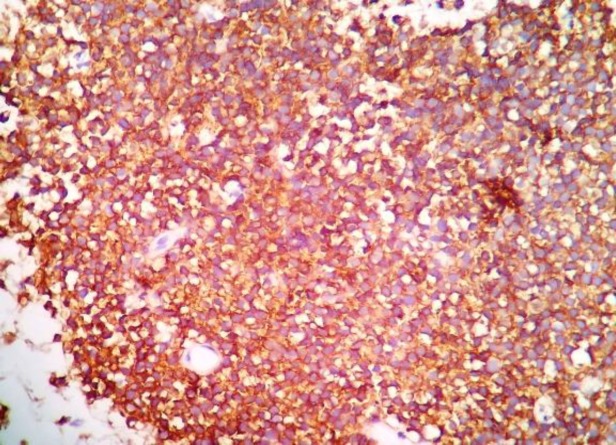
CD99 immunohistochemistry with strong membranous positivity in tumor cells (CD99 × 400)

Brisk mitosis and large areas of tumor necrosis were seen. Immunohistochemically, the tumor cells were positive for CD99 and negative for smooth muscle actin (SMA), desmin and chromogranin immunostains. Weak periodic acid-Schiff (PAS) positivity was also noted (Fig.3). 

A CECT of the chest and abdomen revealed multiple lung nodules with lytic lesions in the dorsal vertebra suggestive of metastasis. Hence, a final diagnosis of EES of the parapharyngeal space with lung and skeletal metastasis was made.

In view of the disseminated disease, chemotherapy was started with vincristine, doxorubicin and cyclophosphamide at standard doses. The patient received six cycles of chemotherapy, and showed improvement in symptoms. Repeat CECT showed persistence of the parapharyngeal lesion with skeletal metastasis, although resolution of the lung nodules was noted. The patient was then given palliative local radiotherapy of 36 Gy in 20 fractions over 4 weeks. However, 1-month post radiotherapy, she presented with complaints of headache, vomiting and medial deviation of both eyes. Subsequent brain CECT revealed an intensely enhancing mass in the left frontoparietal region with mediastinal shift and uncal herniation. Following the CECT brain findings, palliative whole-brain radiotherapy of 25.2 Gy in 14 fractions was given at a rate of 1.8 Gy per fraction. The patient deteriorated further with rapid progression of local and systemic disease. Despite the best medical efforts, the patient succumbed to her disease 1 year after diagnosis.

## Discussion

EES of the head and neck region is a rare neoplasm, and EES of the parapharyngeal space is even rarer. EES is equally prevalent in males and females and predominantly affects adolescents and young adults. Allam et al. reported a median age at diagnosis of 16.5 years and a male to female ratio of 2.4:1(3). Biswas et al. reported a median age of 12 years and a male to female ratio of 2.5:1(5). The most common presentation reported in the literature is a rapidly growing painless mass (3,4,6). The two most important prognostic factors which determine the clinical outcome in a given patient are the age and stage of the patient. The response to chemotherapy is one of the most important prognostic factors affecting disease-free survival (DFS) and overall survival (OS) of patients with Ewing’s sarcoma of the head and neck region (3).

To the best of our knowledge, only three cases of EES of the parapharyngeal space have been reported in the literature (7–9). The first case of EES in the parapharyngeal space was in a 53-year-old male reported by Ng et al. (7). The patient presented with a mass in the right parapharyngeal space with skull base destruction and intracranial extension, and died of the disease 6 months after the initial presentation. The second case was reported by Chaudhary et al. in a 6-year-old male who presented with stridor (8). Imaging revealed soft tissue mass in the right parapharyngeal space with intraorbital and intracranial extension. The patient had a good response to chemotherapy and radiotherapy, and was disease free 10 months post-treatment. The third case was reported by Ramos-Rivera et al. in a 23-year-old female who was treated with chemotherapy followed by autologous stem cell transplant (9). 

She expired 14 months after excision due to an intra-cerebral hemorrhage, and the autopsy revealed no recurrent or residual disease. Our patient had disseminated disease at presentation, for which she was started on chemotherapy and subsequently given radiotherapy to palliate local symptoms. However, due to the progressive nature of disease, she could not be saved. The treatment of EES is multimodal, comprising of surgery, chemotherapy and radiotherapy. However, the parapharyngeal space is difficult to reach and it is difficult to perform surgery most of the times due to the advanced presentation of the disease, anatomic complexity and close proximity to the surrounding structures. Siegal et al. reported that patients with biopsy only or complete resection had significantly better survival rates when compared with patients with incomplete resection (6). 

In a retrospective analysis of 24 patients with Ewing sarcoma of the head and neck region reported by Allam et al. (3), most patients were treated with systemic chemotherapy plus localized irradiation following an initial biopsy. The 5-year actuarial OS and DFS rates were 53% and 30%, respectively, which were slightly lower than figures reported by Intergroup Ewing’s Sarcoma Study (IESS), which is likely due to the large tumor size at presentation (>10 cm in the majority of patients) and low compliance rate to chemotherapy. In a series of 35 cases treated with uniform chemotherapy protocol by Biswas et al. (5), 5-year event free survival (EFS), OS, and local control rate were 55.1±9.2%, 68.3±8.3%, and 74.1±8.5%, respectively, in the whole group. In the largest series of 51 patients with Ewing sarcoma of the head and neck, the most common primary sites included the skull (45%), maxilla (14%) and mandible (12%). The 3-year EFS and OS rates were 74% and 87%, respectively, for patients with localized disease. Patients younger than 15 years had better EFS and OS compared with patients older than 15 years. No difference in EFS and OS could be found when comparing patients with localized disease treated with surgery, radiotherapy or combined surgery and radiotherapy (10).

In the present case, the patient was treated with both chemotherapy and local radiotherapy due to the advanced stage of the disease; however, she ultimately succumbed to her very aggressive disease.

## Conclusion

EES of the parapharyngeal space is an extremely rare occurrence with only three cases reported in the literature to date. Herein, we report the fourth case of EES of the parapharyngeal space. Diagnosis is based on tissue biopsy. Aggressive multimodal treatment should be considered due to the aggressive nature of the disease. It is difficult to comment on prognosis based on four cases. More reports are needed to determine the nature and prognosis of this rare neoplasm.
